# The well-tempered color circle: A chromatic Gestalt

**DOI:** 10.1177/20416695241269314

**Published:** 2024-08-22

**Authors:** Jan J. Koenderink, Andrea J. van Doorn, Doris I. Braun

**Affiliations:** 9175 Justus Liebig University Giessen, Germany; 26657 KU Leuven, Belgium; 26657 Justus Liebig University Giessen, Germany Utrecht University, the Netherlands; Justus Liebig University Giessen, Germany

**Keywords:** color circle, æsthetics, reproduction mismatch, hue discrimination

## Abstract

The “Color Circle” is an important chromatic Gestalt in the visual arts. There is not really a formal equivalent in conventional colorimetry. The fact that the hues can be linearly ordered and that such an order is necessarily periodic was intuited by artists in the early 19th century, but only formally explained by Ostwald and later Schrödinger a century later. As with musical keys, various metrical orders are in common use. Is there such a thing as a “well tempered” order? We consider this an issue for experimental phenomenology. We discuss an attempt based on observations by 30 (nonartist) observers.

According to the Merrian-Webster (see https://www.merriam-webster.com/dictionary/color circle) a “color circle” is

**color circle** noun:

an arrangement of hues in their natural spectrum order (red, orange, yellow, green, blue, violet plus the purples) about the circumference of a circle usually with pairs of complementary hues represented on the opposite ends of diameters.

(First known use 1840.)

Color circles come in many, mutually ontologically distinct, varieties. The first intuitive, but abortive attempt was by Isaac Newton (1704) (see Figure 1 left). The first (intuitive) successful attempt was by Philip Otto Runge (1810), a Danish artist. The first (also intuitive) empirical and largely correct account was by Wilhelm Ostwald (1917). Finally, the definitive (in the formal sense) account is by Erwin Schrödinger (1920a).

**Figure 1.**
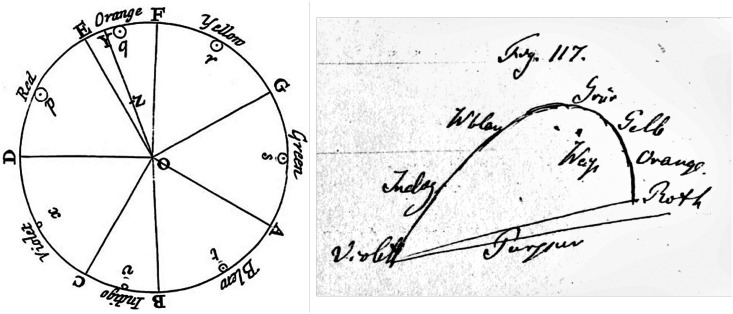
Two historical attempts at the representations of hues. At left Newton’s attempt, perhaps inspired by Descartes’ map of musical intervals. It is more than a “color circle,” since Newton indicates a “centre of gravity.” Note the unequal intervals. At right Helmholtz’s sketch for a chromaticity diagram. The open spectrum locus is closed with a purple line.

There is an ontological cleft between *formal* and *eye measure* accounts. The former are based on colorimetry (starting with Ostwald and Schrödinger), the latter derive from phenomenology, mainly “artistic vision.” (We use “eye measure” instead of “intuitive,” because intuition applies also to the formal and empirical domains).^1^ “Eye measure” stands for “artistic judgement.” Well known (and historically important) instances are Runge (1810) and Albert Munsell (1905). Artistic color circles (Quiller, 1989) that focus on artist’s pigments are also of this type.

Color circles in actual use by designers and artists may be of either variety. Classically, eye measure is all that counts. The increasingly important use of electronic media has changed that. Current “color pickers” (Koenderink et al., 2016) tend to be of the formal variety. A reason might be that the conventional pigments used by artists are not easily parameterized, whereas “electronic colors”—due to mere technical reasons—come by quantitatively parameterized user interfaces—“color pickers” (Koenderink et al., 2016), and so forth.^2^

Here we attempt to adopt a formal structure. We also describe an attempt at a mensuration based on a simple experiment that anybody can conveniently emulate.^3^

## The Formal Geometry of the Color Circle

Colorimetry proper, essentially due to Maxwell (1855) and Helmholtz (1855) over the 19th century is about proximal stimuli and thus lacks a concept of color circle (Figure 1 right). One often sees the boundary of the CIE xy chromaticity diagram (CIE, 1932) proposed as a scientific explanation of the color circle. This is obviously nonsense.^4^ Chromaticity diagrams are two-dimensional projections of the three-dimensional color space, obtained by ignoring intensity. The “spectrum locus” tends to appear as an open convex curve (horse shoe shape), Maxwell called it a “hook.” Indeed, the inventors of colorimetry (Maxwell and Helmholtz) thought of it as the very antithesis of Newton’s color circle. Helmholtz was shocked at the discovery that Green has no complementary in the spectrum.^5^ Indeed, a century and a half after Newton’s circular representation it was discovered to be empirically unsound.

**Figure 2.**
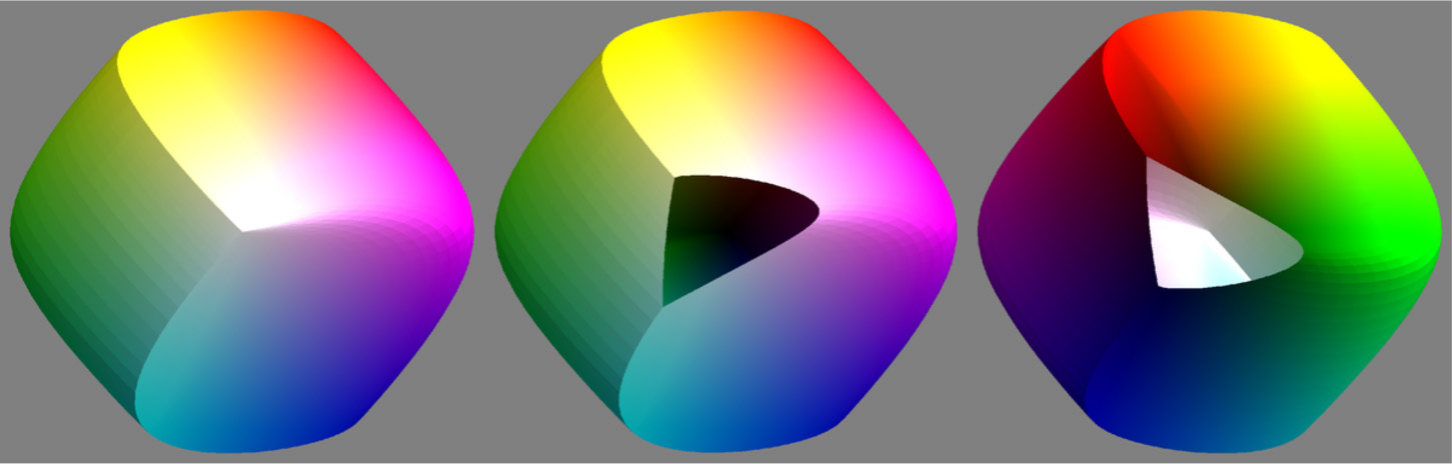
Three views of the color solid from the direction of the achromatic axis. In such views the boundary represents the color circle, for these colors are at the greatest distance from the achromatic axis. The left and center views are towards the white point. In the center and right images small pieces were chopped off with a planar knife, at right angles to the axis. Consider the image at right, which is a view towards the black pole. Note that the shape of the “wound” is the outline at the chromaticity diagram, thus proving that the black point is a conical point. The surface is tangent to the spectrum cone. Next consider the image at center, which is a view towards the white pole. The white point is a conical point where the surface is tangent to the inverted spectrum cone. This is—of course—due to the central symmetry of the color solid.

The formal structure is due to Ostwald and Schrödinger, who considered—in the simplest possible way—the distal stimulus. It dates from the 1910 to 20s and pertains to object colors (distal stimuli), thus escaping the strict colorimetric realm. Figure 2 shows a connection between the chromaticity diagram and the color solid. In formal terms colorimetry applies to the tangent cone of the color solid at its black pole.

The first thing to understand about the colorimetry of object colors is the fact that the full gamut is a finite, convex volume that is centrally symmetric. In contradistinction, the gamut of arbitrary radiant beams is an infinite convex cone. The reason is that diffusely scattering objects cannot scatter more to the eye than what impinges on them in the first place, typically the daylight illumination. Moreover, this body is characterized by an achromatic axis meeting the boundary in a black and a white pole. This again implies the existence of an “equator” of points that are as chromatic as possible. This is the “color circle,” and indeed explains why it has to be a periodic scale.

**Figure 3.**
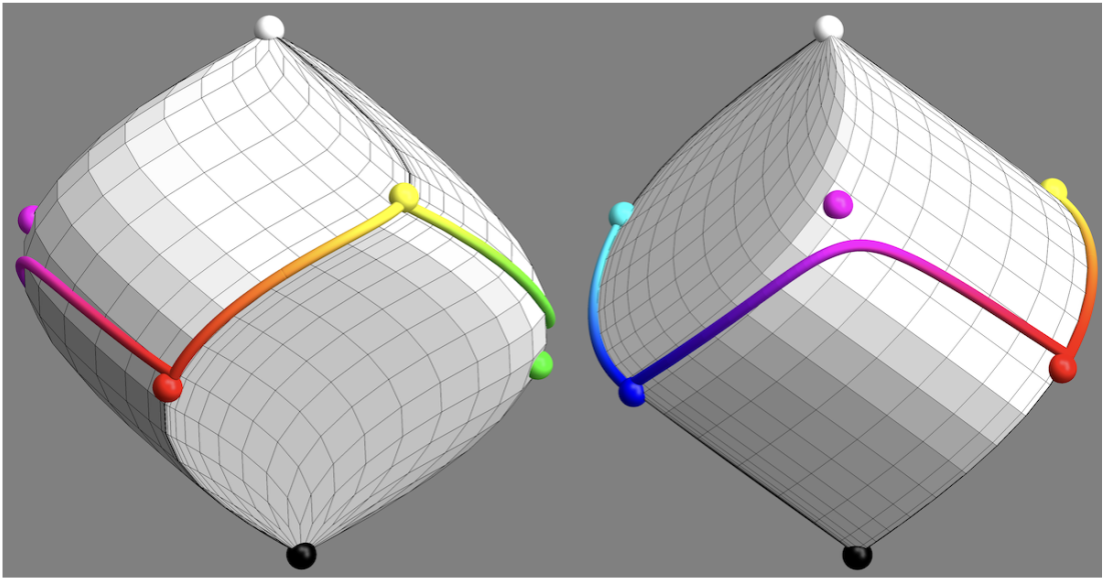
These views are orthogonal to the achromatic axis. Here the color circle appears as the (twisted!) “equator” of the color solid. The points are the vertices of the most voluminous inscribed hexahedron. Note the black–red–yellow–white and white–cyan–blue–black dihedral edges, that are helicoidal curves over the surface of the color solid. (The conical points and dihedral edges are the singularities of the otherwise perfectly smooth surface of the color solid.)

The intuitive understanding of this topological structure is due to Runge (1810). He envisaged the color solid as a perfect sphere. At least he had the main features, finiteness, topology, convexity, and central symmetry exactly right. It was Schrödinger (1920a) who supplied the detailed geometry—more than a century later (Figure 3).

The gamut of object colors is bounded by a smooth—except for local dihedral edges and conical points—surface which is convex and centrally symmetric (Koenderink, 2010).^6^ Thus the osculating cylinder with axis parallel to the black-white diameter touches the surface along a smooth curve (except for local corners). Geometrically this is very much like the equator of the earth’s globe (Figure 3). This curve is the formal color circle adopted by Ostwald (1919a). It was the first instance of a semi-formal color circle in which purple (formally!) appeared on a par with “spectral ” cardinal colors—a major departure from Newton. This breakthrough failed to impress the color science community, given the fact that one may find on the Internet that purple is not a color because it fails a corresponding wavelength. The latter view is even common with scientists (https://www.colorwithleo.com/is-purple-technically-a-color/ and https://www.youtube.com/watch?v=79CIbKGuRLk).

Ostwald was the first person to actually *see* the colors of the actual color circle (Ostwald, 1917, 1919a, 1919b). He may well be the last one too, because one cannot display the colors of the true color circle on a generic trichromatic display. At least not exactly, although quite closely.^7^ So, very few color scientists today have ever experienced the true full colors themselves.

Ostwald had built an inverse spectroscope (based on an invention of Maxwell) that allowed him to produce the “full colors” as he called them. The device can show the colors, but cannot easily be turned into an image display, which is why it is generally ignored. Such instruments are simple to construct with elementary tools. The only optical element required is a prism or grating. It is very much worth the effort, because it allows one to experience instances of Schrödinger’s “ideal colors” and Ostwald’s “full colors.” It is an easy matter to produce essentially perfect realizations of fullcolors (or “semichromes”). One relevant observation is that modern display units are not so bad (of course, the industry does not tell you that), the perfect colors are not that much better than what can be produced by electronic means. Even the latter exceed the gamuts of the natural world or classical art. What more does one want? Although as chromatic as possible, the full colors are not monochromatic.^8^ Rather to the contrary, they scatter *half* of the illuminant spectrum. (“Half” is to be understood in the sense that the spectral boundary locations are mutually complementary.) Ostwald intuited this fact and spoke of the full colors as “semichromes.”

**Figure 4.**
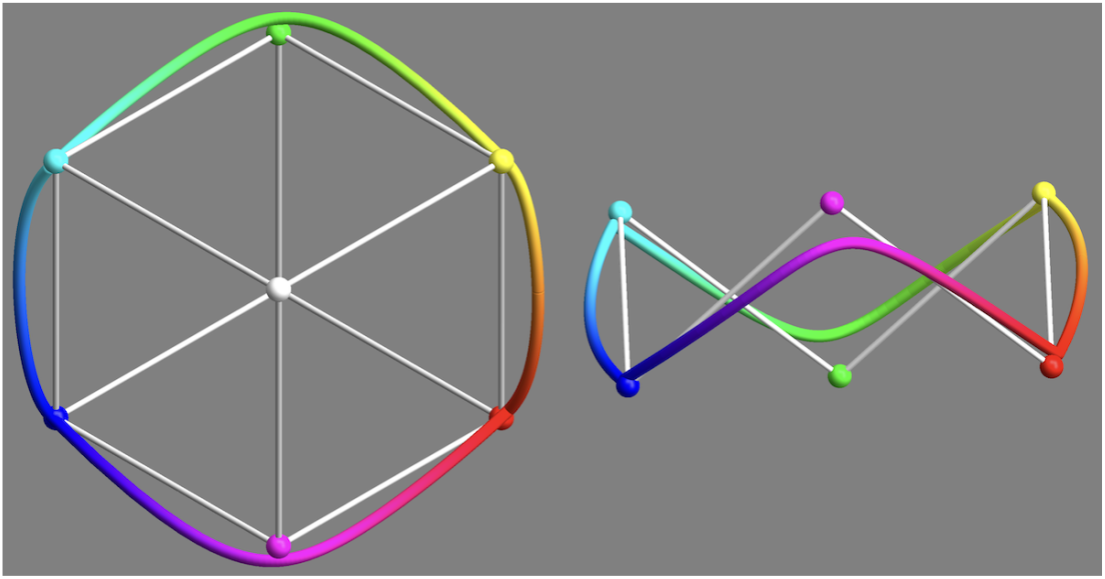
Here one sees the skeleton (the gray rods) of the most voluminous inscribed hexahedron, whereas the color solid has been omitted. The equator of the color solid is a twisted curve that approximately follows as edge progression over the hexahedron. This edge progression red–yellow–green–cyan–blue–magenta–red is the best representation of the true color circle that can be produced on a typical tri-color (RGB) display. In this article, we use a representation that lets the hexahedron appear as a cube.

Generic trichromatic display units cannot generate semichromes (and thus full colors) since they have to approximate the color solid with a hexahedral volume. Ideally this should be the inscribed hexahedron of maximum volume to the color solid (Koenderink, 2010). (The lack of a metric^9^ is bypassed by noting that arbitrary linear transformations leave the ratio of volumes invariant. “Maximum volume”—relative to a reference volume—is well defined.) Some implementations come close, but none is perfect, because the primaries that can actually be implemented are not semichromes. With trichromatic displays the color circle is represented by a closed edge progression. It is the usual red–yellow–green–cyan-blue–magenta–red series implemented by trichromatic displays. It is a general (nonplanar) piece-wise linear curve (Figure 4).

## The Chromatic Edge Progression Over the RGB Cube

The RGB cube is the approximation to the color solid that defines the gamut of colors open to trichromatic display systems. This “approximation” is actually very good Koenderink (2010). There exist almost no object colors in the environment that lie seriously outside the RGB cube. (Of course, all object colors lie within the color solid, a shallow layer covering the RGB cube.)

The edge progression red–yellow–green–cyan–blue–magenta–red is indeed essential in all formal descriptions of the RGB cube. Since it is a closed, twisted, and polygonal arc (Figure 4 right) many descriptions use a polar (or cylindrical) parameterization.^10^ Thus, the curve is either parameterized by arc length (for a unit cube total length 6, usually mapped on the unit interval) or by hue angle (0 ∘ –360 ∘ say). Sometimes the parameterizations are confused because (expressing angles in radians) 2π≈6 —often one wouldn’t notice the difference. Angular measures tend to be mapped on the unit interval too.

## The Circular Representation of the Color Circle

Since the true locus of most chromatic colors is closed, a circular parameterization makes intuitive sense. This applies both to the formal and the eye measure approaches.

**Figure 5.**
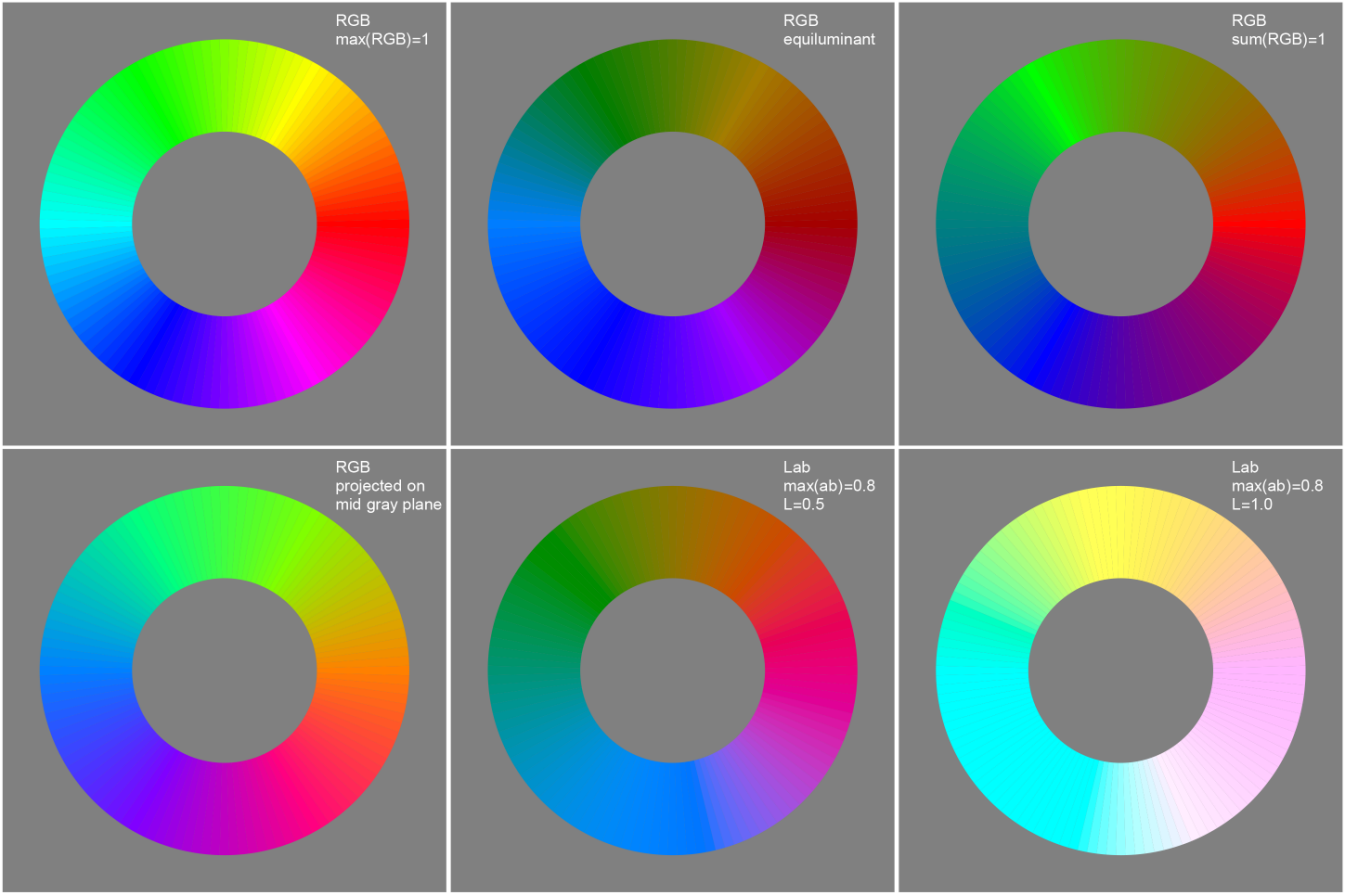
Some attempts at a “well tempered” color circle. Aspects to look for are completeness and vividness of the colors (especially yellows and blues), apparent unevenness in any aspect and the expression of basic colorimetric symmetries. Depending on the application likely contenders are the straight sRGB full colors (top left), their projection on the mid gray plane (bottom-left) and the CIELAB (CIE, 2004) tints (bottom center and right). The latter is the “official” (CIELAB) winner. In this article, we use the RGB structure. It would be the obvious choice of the common user (artists and designers using electronic means). It is adopted in many “color pickers” in graphics applications. Although officially “wrong,” on the work floor (studio) it is “right.” Indeed, other choices would lead to complaints and loss of customers.

In formal approaches one applies some kind of projection to obtain a circular (or also hexagonal) arrangement (Figure 5 top left). In the eye measure approaches, a circular arrangement is often proposed as a matter of course, without further justification.

Here the desire to obtain a planar “overview of all colors” often settles the choice. The human mind has major difficulties with the third dimension. This is also the reason for the popularity of chromaticity diagrams, mostly with unfortunate consequences.

The “color circle” in the restricted sense is only one-dimensional (albeit closed) thus one can really wrap the mind around it. This applies to chromaticity diagrams too, where most people concentrate on the spectrum locus. The natural periodicity runs against the notion that one “sees color by wavelength,” for the linear interval is simpler than a closed loop. This still dominates many pop-science accounts of color and is a rich source of misconceptions. A common one is that “purple is not a color, because there is no corresponding wavelength.” Up to the reader to make sense of that.

One way to conceive of the color circle is as an almost irresistible Gestalt. It beats the spectrum locus anytime. Newton’s abortive color circle was liked as a strong Gestalt and intellectuals welcomed the spectrum as a “scientific” justification of it.

## Desirable Properties of Color Circles

From a formal perspective a color circle is nothing but some approximation to the Schrödinger account. One would certainly like to preserve the essential features, which are at least finiteness, convexity and central symmetry. Perhaps one would also like to see a reflection of the Schrödinger (following Ostwald) distinction between four types of “ideal” spectral albedos. “Ideal” albedos (Bouma, 1946) map on the boundary of the color solid. They have albedo zero or one with at most two transitions Schrödinger (1925). This implies a division of the color circle into four natural parts.^11^ Such a division was also proposed by Ewald Hering (1878), based on phenomenological (pure eye measure) arguments. As with many objects, there is a close correspondence between eye measure and formal definitions. This is remarkable, because there is no scientific “reason.”

These features should also be reflected in the divisions of color circles into discrete cardinal elements. The number of such element should be *even* because of the central symmetry. It should also be a multiple of three because three edges of the RGB-cube meet at the black and white poles, or—more abstractly—because human vision is trichromatic. Finally, it should be a multiple of *four*, because of the Schrödinger spectral albedo types. Thus, the simplest discrete representation has *twelve* ( 3×4 ) steps. This may be the most useful discretization, but one frequently meets with finer subdivisions. The practical limit is less than about 48 steps because most people have a hard time to sort such a structure—when scrambled—by eye (Koenderink et al., 2019). A 24-step color circle is useful (most people can sort it), whereas a 60-step one is severe overkill (almost nobody can sort it).

Such structures often conflict with other desirable features. For instance, Ostwald (1917), who was an outspoken defender of the decimal system and pushed it where he could, originally started with a division in ten (neither a multiple of three nor a multiple of four). He changed that at a later stage. Albert Munsell (1905) based his system initially on a five-way (an odd number) division based on eye measure. He essentially merged the cyan and green vertices that many observers indeed phenomenologically merge.

Munsell’s choice was due to the fact that the basic RGB cube edge progression fails to look “uniform” to the artistic eye. One easily checks that through a cursory look (Figure 5). Thus (subjective) “metrical” properties may also play a role. Implementing this is likely to destroy formal symmetries, such as central symmetry. This again leads to adjustment of relations such as complementarity (Koenderink et al., 2020).

All considering, the structure of the color circle is only partially to be based on formal color theory. It is a matter of choice, dictated by the particular interest or need.

## Various Attempts at Color Circles

Our title is obviously patterned after Bach’s *Das Wohltemperirte Clavier oder Præludia, und Fugen durch alle Töne und Semitonia, …*. (The concept “*wohltemperiert*” might be due to Werckmeister’s (1686) “*wohltemperierte Stimmung*.”) Is there such a thing as a “Well Tempered Color Circle?”

It is likely that Newton’s attempt at a color circle was inspired by René Descartes’ concept of the musical intervals in the octave. The relation between colors and melodious sounds has a long and varied history, but one may well doubt its validity. We consider it an issue of experimental phenomenology, rather than mere speculation.

We show the basic RGB edge progression as a circle in Figure 5 top left. This retains important symmetries, perfect central symmetry and approximate bilateral symmetry about the teal-orange axis (Koenderink & van Doorn, 2021b). It contains all hues in their most vivid form as constrained by the trichromatic display (roughly sRGB).

These desirable features are perhaps offset by a number of nonuniformities that appear at first blush. This is why color scientists (not so much users) have attempted to iron out the nonuniformities. On the whole this has met with little success (see Figure 5, most of these do not work very well, abeit for diverse reasons).

In our view, there is no such a thing as the “perfect” color circle. From an applications perspective the choice is settled, the generic color pickers use the RGB edge progression in some way or other. This retains the formal symmetries, but leads to a phenomenologically nonuniform color circle. We feel that the advantages prevail and that uniformity will necessarily come at the cost of various disadvantages. Uniformity—no matter how one defines it—is an impossible goal. For instance, any attempt to render blue and yellow “equibright” (in whatever formal sense) will destroy the essential phenomenological nature of these hues. It may be best to accept such nonuniformity as one of the characteristics of color.

In this article, we use the basic RGB structure, which is not only attractive from a formal perspective, but also widely used by artists and designers using current electronic tools.

## The Empirical Mensuration of the Color Circle

### Methods

How does one go about the construction of a “well tempered” color circle?

One first thinks of psychophysics (Fechner, 1860). The classical way to calibrate the color circle would be to find discrimination thresholds over the full circle. Such experiments are done using split-field presentation on a uniform background (MacAdam, 1942; Wyszecki & Stiles, 1967). In strict colorimetry this has led to the understanding that humans discriminate over 10-million colors over the full color space and about 50 to a 100 over the spectrum locus.^12^

One wonders how to square this with the fact that luxury boxes of dry pastels (Rembrandt, 2023)—whose pigments are generally not “mixed”—contain rarely more than a few dozen (up to about 200) pieces. There is a gap of many orders of magnitude.

One reason is that artists tend to act differently from psychophysicists. Artists and designers work with structured backgrounds and rarely use split-fields. They tend to be more interested in obvious contrasts than differences one has only a three-to-four chance of detecting. One looks at a spot, then looks somewhere else and attempts to reproduce the patch that was seen. This may (or may not) be tested by looking back and forth a few times. Emulating this in the laboratory yields a statistical insight in the “reproduction mismatch.” In previous experiments, (Koenderink et al., 2018), we found that the number of colors that can be reproduced is of the order of hundreds, perhaps up to a thousand. In many applications, it is the reproduction mismatch, not the discrimination threshold, that counts.

The psychophysical limits are determined by physical factors, that is, the structure of electromagnetic radiation (Planck, 1901; Born and Wolf, 1959) and the anatomical and physiological structure of the visual system (Roorda & Williams, 1999; Dacey & Packer, 2003; Kim et al., 2023). The reproduction mismatches do not necessarily reflect such limits.

When the system is not limited through the essential constraints they are limited by psychological, behavioral factors. From a biological viewpoint one looks for causes in terms of optical factors related to vital interactions of the observer with the Umwelt (Koenderink & van Doorn, 2017; Koenderink, 2018; Koenderink et al., 2021a). In the case of color metamerism limits the value of color for recognition, whereas it leaves its value for discrimination unscathed. Thus one expects the behavioral limits to be task dependent.

#### Paradigm of reproduction mismatches instead of split-fields

**Figure 6.**
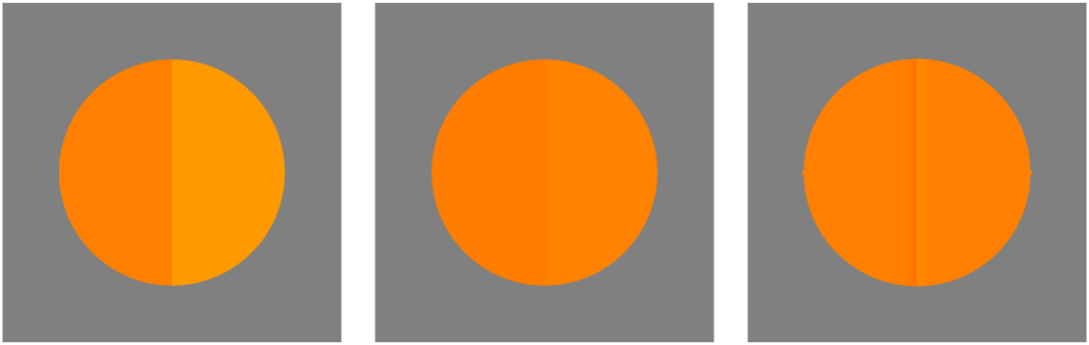
The split-field paradigm. At left the difference is obvious enough that most people feel they are comparing two colors. At center the difference is much smaller (unfortunately, we can’t make it small enough in print). In the limit (near the “color discrimation threshold”) we don’t have the experience of “comparing two colors” at all. Instead, we feel that we are *detecting an edge*. We also feel that detecting edges is very different from comparing colors. At right the two colors left and right are *identically the same*, but we added a fake edge (yielding a “Craik–O’Brien–Cornsweet illusion”). Are the center and right cases experientially different? Not to us. In case you agree (not likely many vision scientists would agree, there is too much established fact at stake!), the split-field threshold does not really address “color discrimination,” but rather “edge–detection.” Although the difference might seem merely “academic,” we feel it is a conceptual game-changer.

In this experiment we avoid the “ideal” case of the classical split-field presentation (Figure 6). It is generally taken as a fact that in the split-field presentation the observer compares the colors that fill the left and right hemidisks. We seriously doubt that. Near threshold our experience is that we do not see the left and right colors as different at all, although we still perceive the edge between them. Thus, we respond on an edge-detection criterion, but not such a thing as a “color difference.” That is why we change the task to what we call a “reproduction task.” We used that method before (Koenderink et al., 2018) and were happy with the results, which were really different from the conventional findings.

In a previous experiment, (Koenderink et al., 2018), we could only investigate the interior volume of the RGB-cube, staying (for technical reasons) well free from the boundary. Here we extend this to the RGB-fullcolor locus. In the past, we also experimented with sorting color circles and found that sorting errors start at circles of about 30 steps. This yields a rough estimate of the reproduction mismatches to expect.

**Figure 7.**
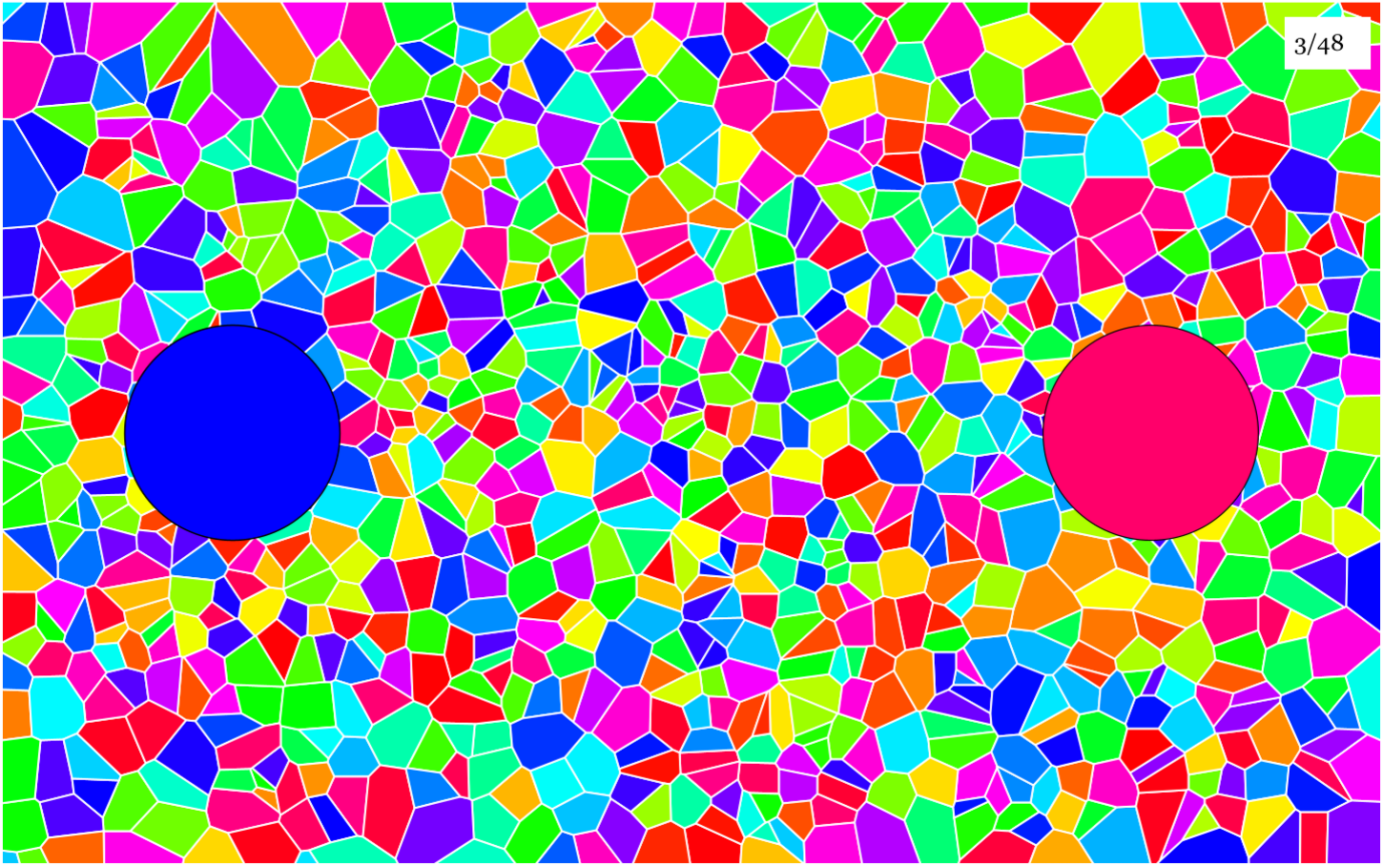
A screen dump from the experimental paradigm. The random background contains all colors from the color circle in even proportion. Its structure changes gradually over time. There are two patches, at left (a blue patch) a fixed fiducial hue, at right (a purplish red patch) a variable hue. The participant has the task to change the right-hand hue to match the fiducial. During manipulation—using the keyboard arrow keys—the fiducial is removed. Thus the matching is on the basis of a short-term memory color. The difference with the psychophysical split field presentations is highly relevant.

In the task used in the experiment presented here (Figure 7) the reproduction mismatches are likely to significantly exceed discrimination thresholds. That is because the conditions for a valid “discrimination threshold” (uniform background, simultaneous viewing, etc.) are seriously violated, each particular violation most likely increasing the threshold.

## Experiment

The experiment focuses on the reproduction mismatches. The “reproduction mismatch” is a concept that lacks a precise definition. It is supposed to capture the generic practice of users in art and design. This rules out the generic psychophysical setups. It requires busy backgrounds,^13^ no common boundary of the two patches to be compared and minimal instances of simultaneous vision. Most comparisons occur asynchronously involving eye movements, distinct “looks.” Of course, such notions need to be made precise in a formal experiment (next section).

### Methods

Participants had to reproduce a fiducial patch on a busy background. As they adjusted the approximation, the fiducial was removed from the display (thus revealing the background). Thus they made comparisons by looking back and forth, and had to rely on very short-term visual memory.

We used 48 fiducials, regularly distributed over the RGB color circle. Since the total length of the color circle (edge progression) is six, they were spaced by a distance 6/48=0.125 . The resolution of the adjustment was much better, half a degree, that is, 1/720≈0.0014 (resolved by the hardware).

In pilot studies, we find that participants reach high levels of precision at the task. The settings and the fiducials have rank correlations (Kendall τ ) in the 0.99 range.^14^

#### Display

The setting was chosen to be as close to a generic user profile as possible. So we present the stimuli on an Apple notebook LCD screen, using the generic Apple LCD profile. It involves a gamma curve of the sRGB-type ( γ=2.4 ). The CIE xy chromaticity coordinates of red, green, and blue are:

**Table:** 

Red	x = 0.6588,	y = 0.3338
Green	x = 0.3204,	y = 0.6137
Blue	x = 0.1505,	y = 0.0527

The chromaticity of the white point was x=0.3126,y=0.3291 . The luminance of the (white) screen was $214.\;\mathrm{cd}/m^{2}$ .

Viewing was informal, binocular at convenient reading distance in a darkened room. It seems unlikely that minor variations of this (in the range of “typical user interfaces”) might make much of a difference.

#### Participants

We tested 30 observers, the majority (17) were students of the university of Giessen, who were compensated for participation either with credit points or money. The other participants were lab members with different expertise with respect to color perception. Participants (77% female, 23% male) were naive to the purpose of the experiment. Ages ranged from 19 to 75 (quartiles [21, 26, 33]). All viewed stimuli binocularly, if necessary wearing personal corrections. The screen was viewed in an informal setting (although the room was dark) from convenient reading distance. All were tested with the 24-plate edition of the Ishihara test (Ishihara, 2018).

### Results

**Figure 8.**
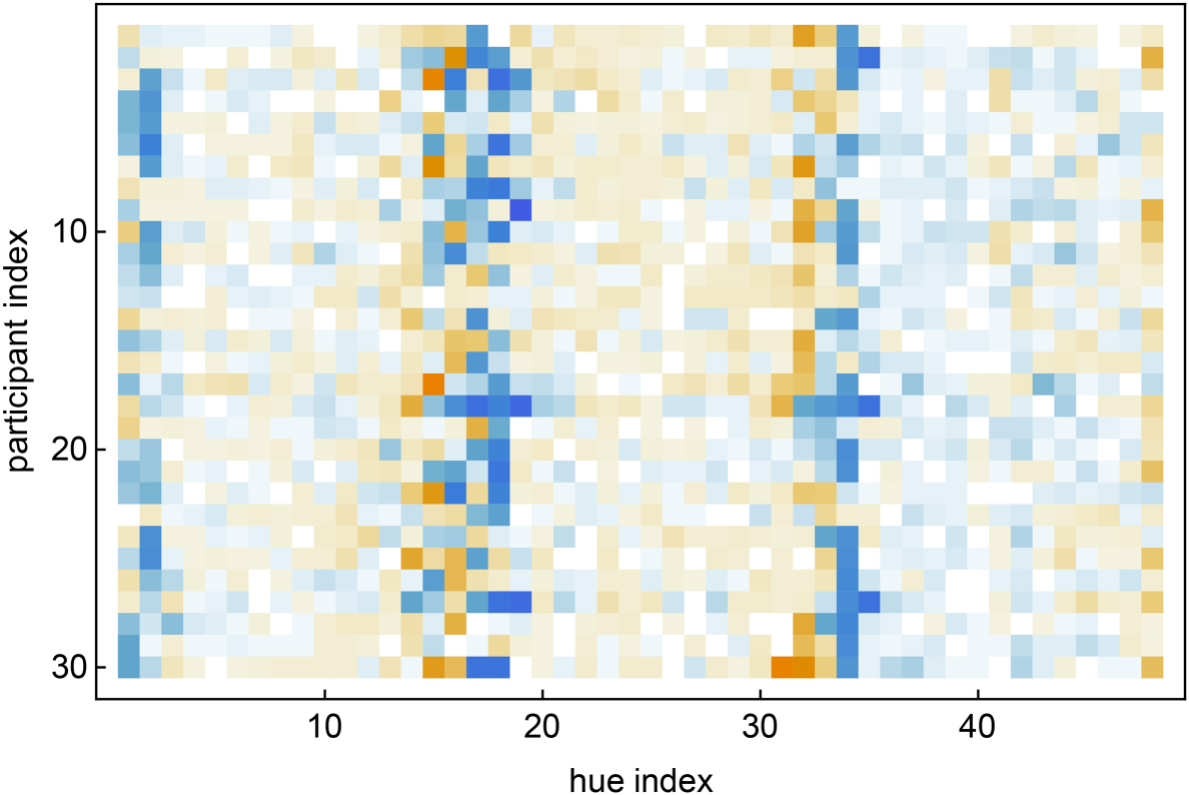
The raw data from the experiment. (Bluish negative, orangish positive, white zero.) The reproduction mismatches are non-uniformly distributed along the hue dimension. There are 1440 ( 30×48 ) reproduction mismatches in total.

**Figure 9.**
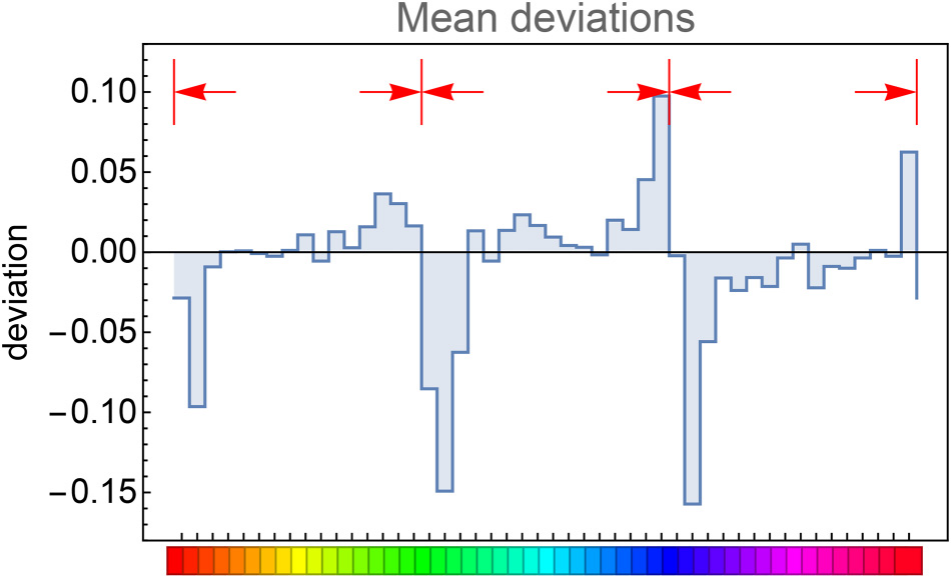
The ensemble mean of the deviations (raw data). The red arrows indicate the direction of the deviations.

**Figure 10.**
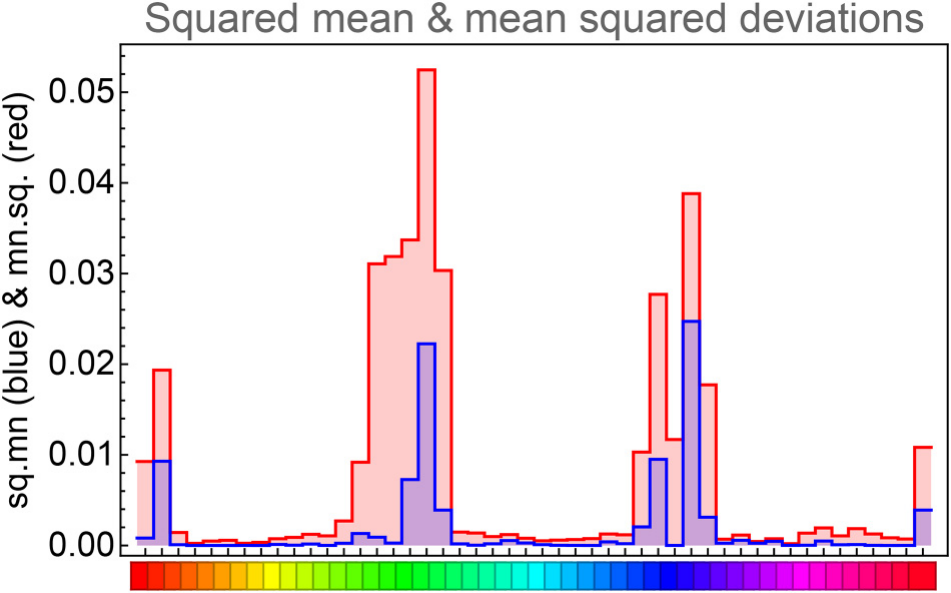
The square of the ensemble mean (bluish) and the ensemble mean of the square of the deviations (reddish).

This session took a little over half an hour. The raw data consists of the reproduction mismatches (Figure 8). For about 40% of the hues the ensemble average (Figure 9) deviates from zero (according to a sign test at the 5% level). The ensemble average reveals a trend to be attracted by the red, green, and blue hues (locations 0, 16, and 33). The average deviations are relatively smallish. In Figure 10, we compare the square of the ensemble average with the ensemble average of the squares of the deviations. The ratio of the totals is about 1:4. The two distributions are clearly related (correlation about 0.8). The effective data used in the analysis are the standard deviation of the reproduction mismatches.

There are two ways to get at the standard deviation.

**Figure 11.**
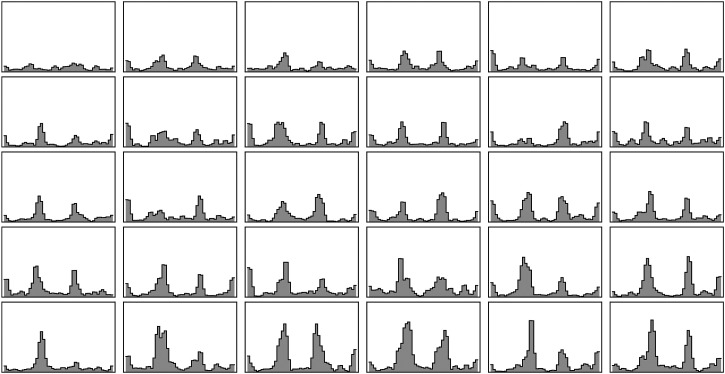
An overview over all participants (sorted by order of the highest peak value). The data are slighly smoothed along the hue scale. These graphs are all on the same scale. Thus the participant shown at top left is “the most precise” and also most uniform over hues.

One way applies to a single observer. Ideally one would repeat setting numerous times. However that was out of the question due to practical constraints. An approximation involves minor spatial smoothing. One squares the errors, smoothes the pattern over the color circle and draws the square root. This will work when the standard error varies gradually, which is a priori likely. We use a Gaussian window and do the convolution computation via fast Fourier transform, which automatically takes care of the periodic boundary conditions. All results are shown in Figure 11.

**Figure 12.**
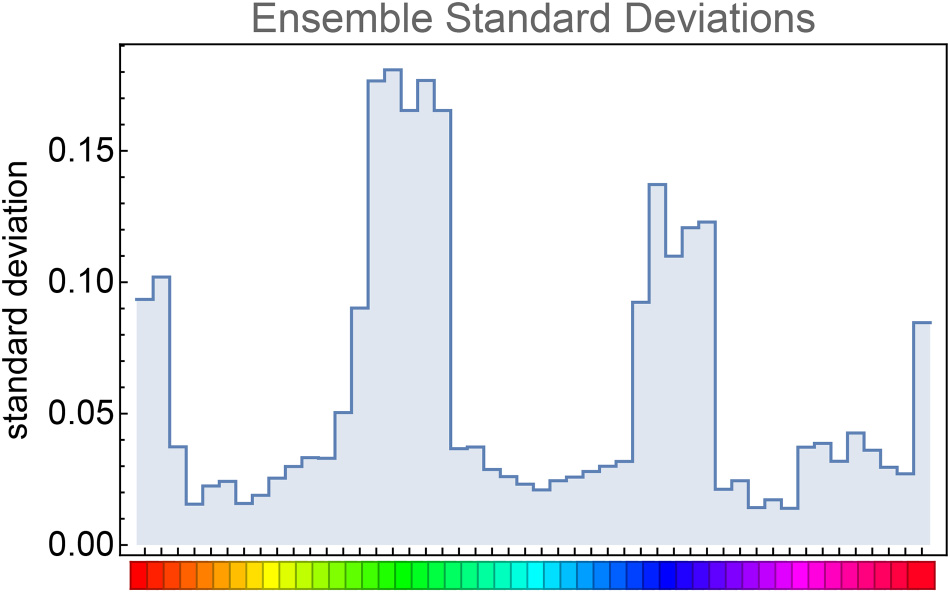
Ensemble data need not be smoothed over hues. Here we derive the standard deviations over all participants (the columns of the data matrix shown in Figure 8).

Another way applies to the ensemble of participants. Here one computes the standard deviation at a single fiducial location over the full ensemble (Figure 12).

These methods (smoothing over hue or ensemble mean) turn out to yield essentially the same results. Of course, these two methods may also be combined.

### Analysis

As a first observation we find that the participants are qualitatively and largely quantitatively rather similar. All correlate better than 0.28 with the median. The median correlation with the median is 0.81, the interquartile range 0.71 to 0.84, full range 0.28 to 0.91. Such numbers somewhat hide real interindividual differences: the range of correlations between individuals is − 0.11 to 0.92, interquartile range 0.49 to 0.75, median 0.64.

**Figure 13.**
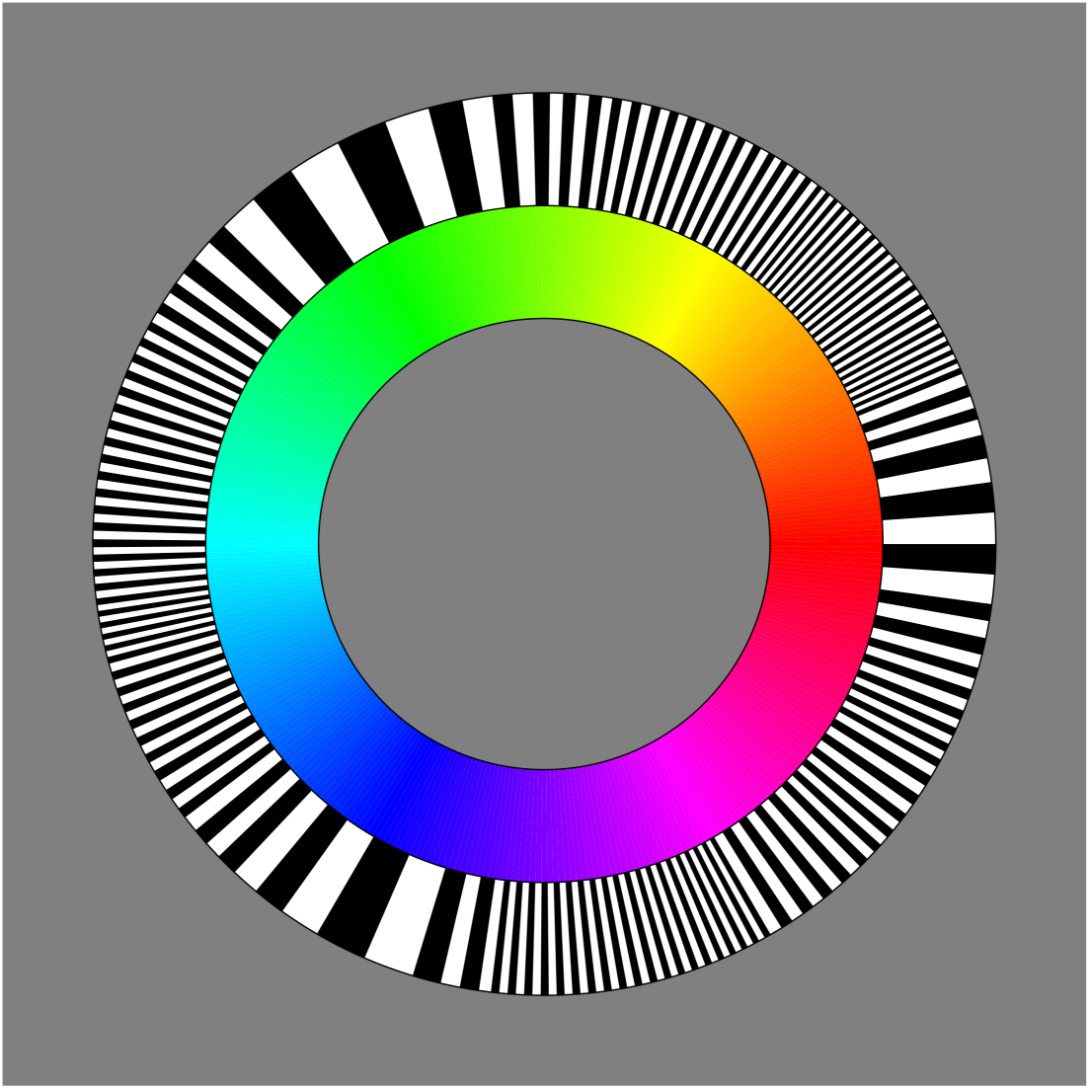
Empirical mensuration of the RGB color circle. This is from the median data of all participants. However, single participant data collected over a 35-minutes session yield about the same results. The resolution has been picked in order to obtain a good visualization, it does not reflect the actual fuzziness. The best resolution is between the RGB primaries, thus cyan (green–blue), magenta (blue–red), and yellow (red–green) where the nature of “between” varies as shown by the smallest black and white stripes. Should we move the hues to force “uniformity?” This would be at odds with the conceptual symmetries. In our view, it makes more sense to accept the nonuniformity as part of the essential structure.

The ensemble standard deviations (Figure 12) reveal three modes at red, green, and blue hues. In Figure 13, we show how this works out over the color circle. The distribution is very uneven.

In a more detailed analysis, we focus on possible differences. Therefore we extract the modes, noting location, halfheight-width, and height. Since the modes are so clearly articulated it is easy to extract these from the data.

**Figure 14.**
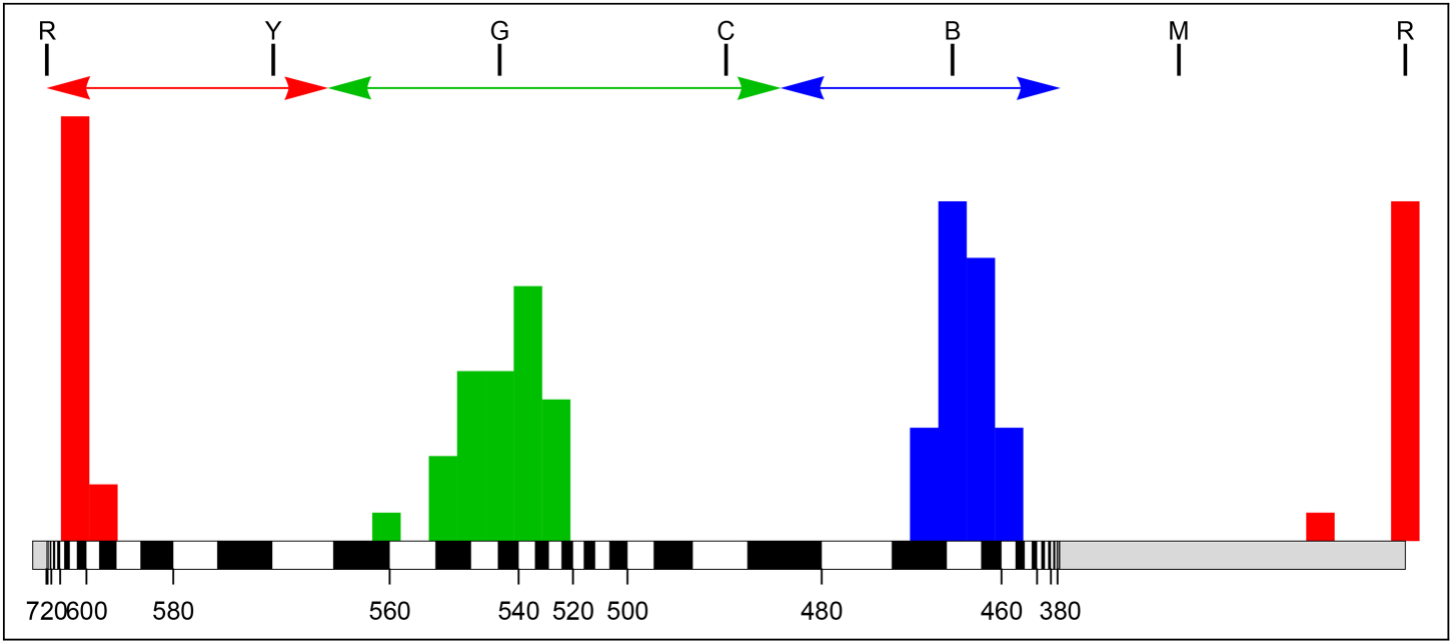
The locations of the modes for all participants. The double-arrows indicate the spectral ranges of the optimal set of spectrum-tricut primaries. The locations of the cardinal colors (as close to the actual ones as the LCD primaries permit) are also indicated. The location of the empirical modes are close to the red (674 nm), green (545 nm), and blue (469 nm) cardinals, whereas the best hue resolution is in between the peaks, at yellow (570 nm), cyan (487 nm), and magenta ( $\bar{545}$ nm).

We find that the locations of the modes differ very little between participants (Figure 14). Although the locations are quite well defined, small differences might turn out to be of interest in the context of some investigation.

The heights and halfheight-widths of the peaks are strongly correlated, so we may regard either one as standing for the other. Here we find differences. For about two-thirds of the participants, the green peak is the highest with the blue one next and the red one lowest (19 cases of GBR). However, in a few cases the blue peak is higher than the green one (five cases of BGR). There are also a few rarer cases, RGB, GRB, and BRG were each scored twice.

**Figure 15.**
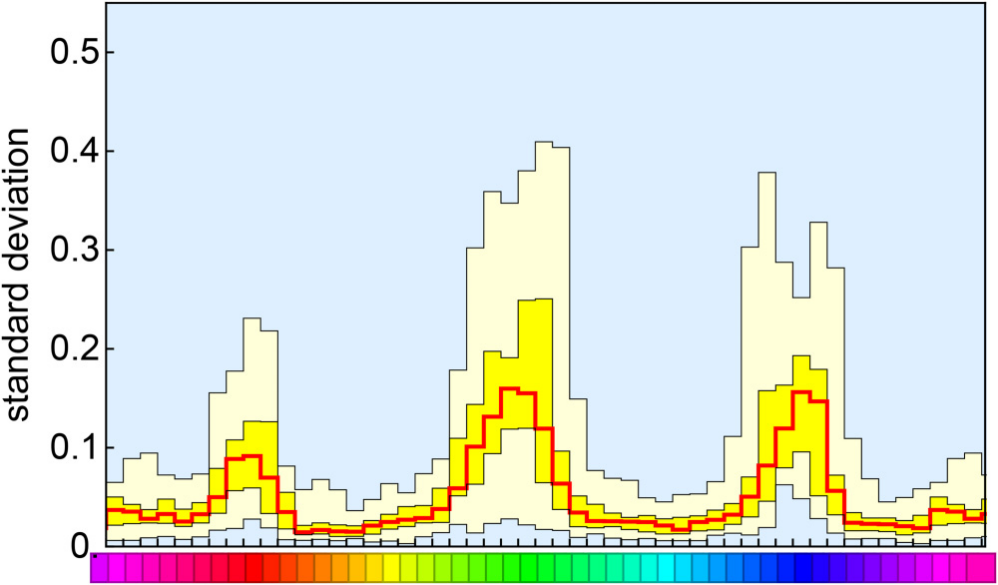
Quantiles of individually smoothed data over all participants. Range pale yellow, interquartile range yellow, and median red.

Figure 15 shows a general overview in form of quantile levels for the whole group of participants. These where obtained from the slighly smoothed individual estimates. The median may be the more important feature, but this representation also reveals something about the interindividual differences. Of course, these are at least partly due to individual tolerances with nonvital risk-taking. One has to reckon with a variation up to a factor of two.

#### How many distinct hues can be reliably distinguished?

How many hues are distinguished along the color circle? We may guess this from previous work (Koenderink et al., 2019). Most people readily sort a 24-step scrambled color circle, but already with 32-step color circles one or more errors occur. Thus one guesses “a few dozen.”

The present data allow a numerical estimate. We have data in 48 bins, thus the bin width is Δ=6/48=0.125 in terms of the RGB-cube edge-length. In each bin i∈0…47 , we have a standard deviation estimate $\sigma_{i}$ . For a 95% criterion, the discernible steps should be $\delta =2\sqrt{2}{\mathrm{erf}}^{-1}0.95\approx 3.92\sigma$ apart. Hence the bin contributes Δ/δ discernible steps. Adding up the contributions of all 48 bins we obtain a total number of discernible steps $N=\sum_{i=0}^{47}(\Delta /(3.92\sigma_{i}))$ .

We may do the calculation for all participants separately. The median is N=58 , the interquartile range 48–64, the range is 34–95. For a 48-step color circle one would expect an average of three sorting errors, which seems about right.

Of course, such a single number hides the fact that the distribution of resolution is very nonuniform. The resolution is lowest near red, green, or blue, whereas it is highest near cyan, yellow, or magenta. However, a single number of merit is frequently usful, as when one compares color blind with generic observers.^15^

The 58 steps imply about 10 steps along an RGB-cube edge-length. Raising this to the third power should yield a rough estimate of the number of discernible RGB-colors: almost a 1,000. This is in agreement with earlier estimates (Koenderink et al., 2018). It is orders of magnitude below numbers commonly suggested in the popular literature, but it agrees with hands-on experience of designers and other such professionals.

### Observations from Further Analysis

When can it be said that an observer can “discriminate” two hues? Here one has to introduce some conventional criterion. Using the empirical standard deviations, we can ascertain that the expected confusion is minor. For instance, one sets the criterion at an overlap of 5% of the confusion distributions. This then implies a separation of 3.92⋯ times the standard deviation.

**Figure 16.**
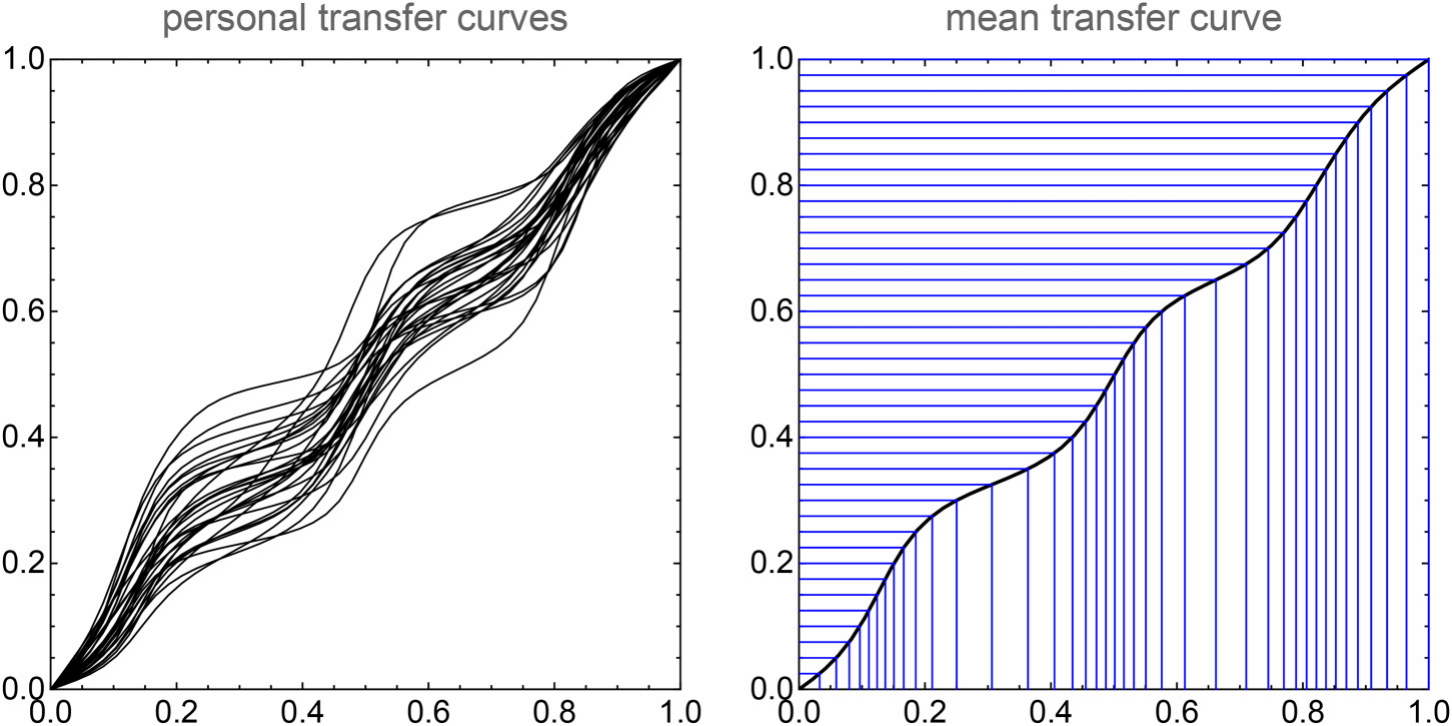
The transfer curves for mensuration of the color circle, these even out the empirical nonuniformities seen in Figure 13. The scale is the conventional hue parameter ranging from zero to one and with red at zero. The mean is used to construct the well-tempered color circle shown as in Figure 17. Note how different the individual transfer curves are! Most of the variation is interindividual. The curves are well reproduced if one repeats a single observer. Any single representation is likely to look uneven for some group of individuals.

**Figure 17.**
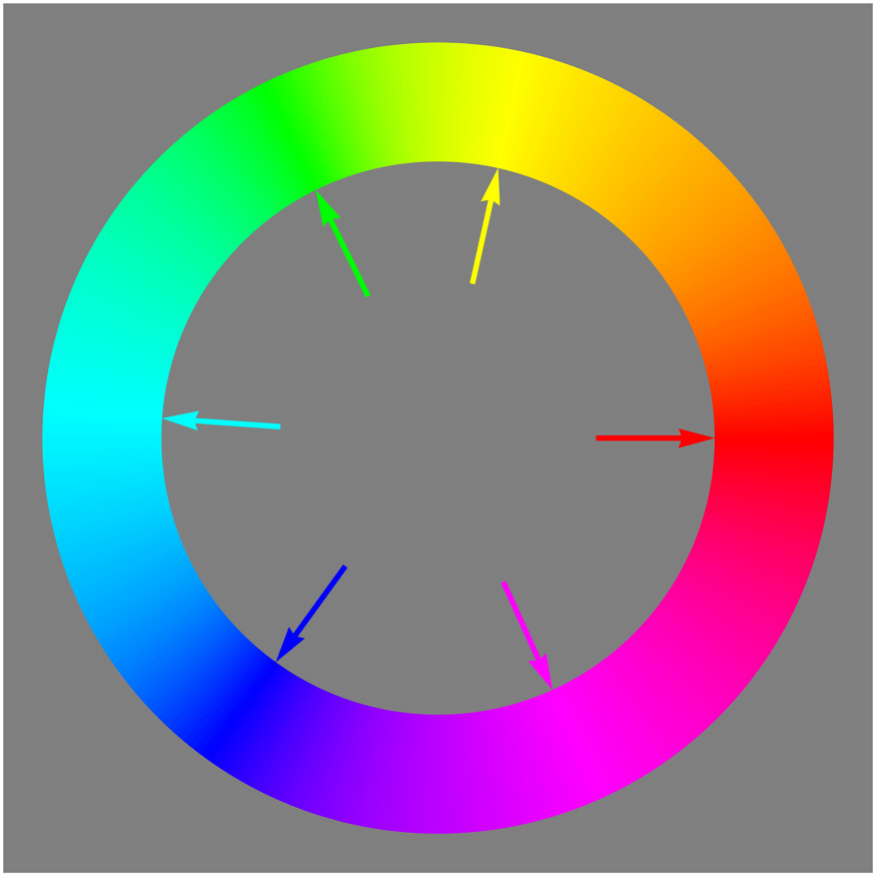
The “well-tempered color circle” for the ensemble mean.

In Figures 16 and 17, we show an attempt at a “well-tempered color circle.” The interpolations are based on the ensemble median. In such a presentation the yellows–oranges and teal–blues are extended, whereas especially the greens are contracted. In our perception, it does not look especially “better” than the raw RGB-circle (Figure 5 top left), although certainly better than the other contenders shown in Figure 5. It certainly looks very different from the raw color circle though.

Note that this type of well “tempered color circle” carries exactly the same colors as the original RGB one does, only the distribution is different. Thus, it should not be compared to the frequent attempts that adjust the colors (e.g., by enforcing equiluminance).

## Conclusions and Discussion

We proposed and tested a quick and effective way to measure the hue resolution over the color circle for individual observers with the paradigm of reproduction mismatches. One obtains informative data in the span of half an hour to three-quarters of an hour.

Such data allow one to construct a personal “well-tempered color circle.” Figure 17 illustrates an attempt based on the median data for our group of observers. Repeating this for individual observers yields qualitatively similar but quantitatively different results (Figure 16 left). The color circle shown in Figure 17 looks quite similar to those by Griffin & Mylonas (2019), based on a categorical metric.

The overall mean is perhaps of interest in the design of “color organs” (Caswell, 1980; Franssen, 1991; Ox & Britton, 2000; Wilfred, 1947). Would Bach have liked it? Sadly, there is no way to find out.

One thing that perhaps strikes one at first blush is that a “summary account” might say the circle is dominated by approximately yellow, red, and blue sectors. Of course, “Yellow,” “Red,” or “Blue” stand for generic color families here. For the well-tempered circle the sector angles appear roughly similar to us.

**Figure 18.**
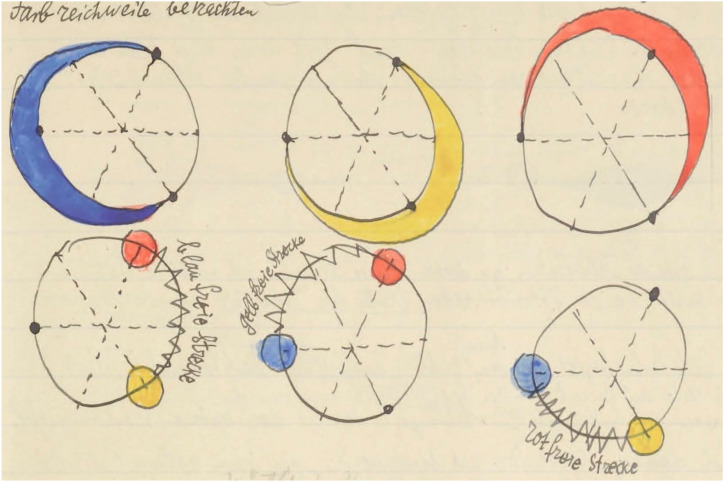
The structure of the color circle according to Paul Klee (2023) is a tritone red–yellow–blue circle. Contributions from red, yellow, and blue each come from two-thirds of the color circle. Klee means the “ideal” distributions in an affective, not an optical sense.

Perhaps this explains the common account (Figure 18) found with artists. The artistic presentation of the color circle is almost always based on red, yellow, and blue. In extreme cases like the Dutch “De Stijl” movement, which was design-oriented in an especially purist way, these were the only colors the artists permitted themselves. They were augmented with the achromatic white, gray, and black. Theo van Doesburg (1925) saw a “New World Order” springing from the use of such purism. The Bauhaus Kurs taught by Paul Klee (2023) is less purist, but the topology of the color gamut is fully based on the red–yellow–blue triad. It must have been the overal Gestalt of the color circle as experienced by generations of arists.

This artistic persuation has never been understood in regular color science. Indeed, yellow is only a very narrow range in Newton’s spectrum. The overall Gestalt of the spectrum is red–green–blue with yellow and cyan as mere boundary elements. The same applies to the Schrödinger/Ostwald “true” color circle. (However, the pass band for the best yellow object colors is very broad, it contains all wavelengths except for the very shortest ones.) The artistic choice of the red–yellow–blue triad seems odd, yet it can hardly be disregarded. One tacitly feels that it is a result of the fact that artists are no scientists. That may be, but they know how to look whereas many scientists look with their ears (Koenderink et al., 2015).

From our construction of the well-tempered color circle, the artistic presentation becomes readily acceptable. It makes some “æsthetic sense.” We discovered the basic importance of the red–yellow–blue triad in a recent study on color harmony (Koenderink et al., 2021c), based on a large group of naive (nonartist) participants. That result also fits in the current context.

**Figure 19.**
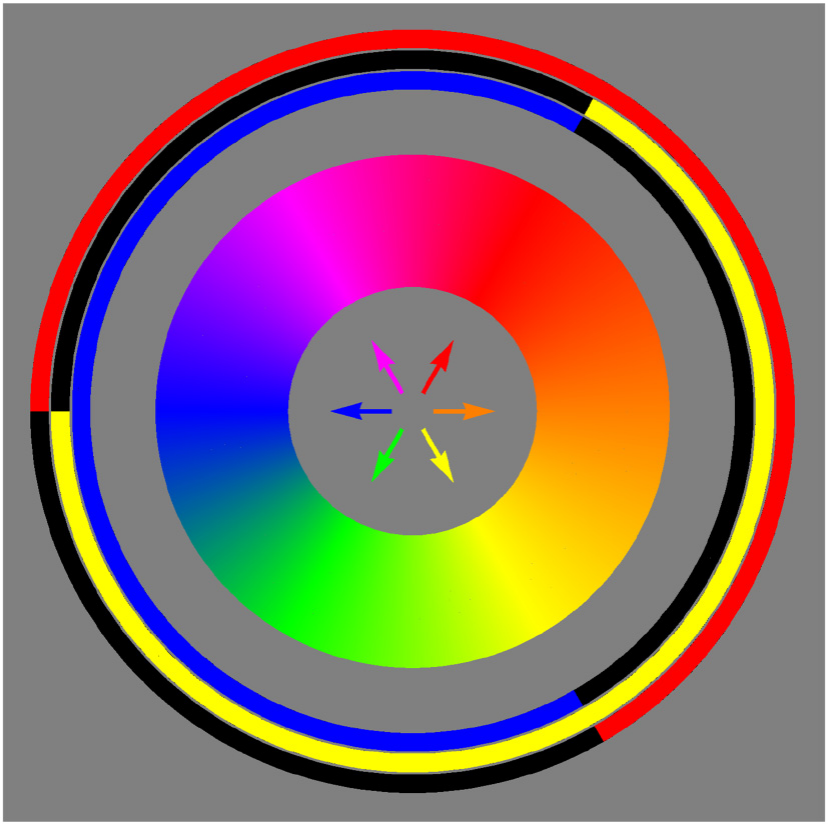
The Klee color circle is rather different from Figure 17. Especially the “yellow” range is widely expanded. Ecologically, it makes some sense, since the bulk of colors of organic nature is in the yellow–red range.

Figure 18 is meant as an instructional aid in “how to see the color circle.” (Compare Figure 19, plotted in our standard format.) At top left you see the *Farbreichweite* (meaning extent of hue). The extents for red, yellow, and blue are indicated as stretching over two-thirds of the color circle. The zig-zag indications marked *gelb*– (yellow), *rot*– (red), or *blau*– (blue) *freie Strecke* (meaning absence of yellow, red, or blue) cover each a third of the color circle. Note that purple must be located in the *gelbfreie Strecke* between blue and red, green is located in the *rotfreie Strecke*, between blue and yellow, and orange is located in the *blau freie Strecke* between red and yellow. (This suggests that the system is intuited on the basis of experiences with subtractive color mixtures.) The diagram perhaps might help you (like it did Klee’s students) to see the color circle in terms of red, yellow, and blue. Then try Figure 19.

**Figure 20.**
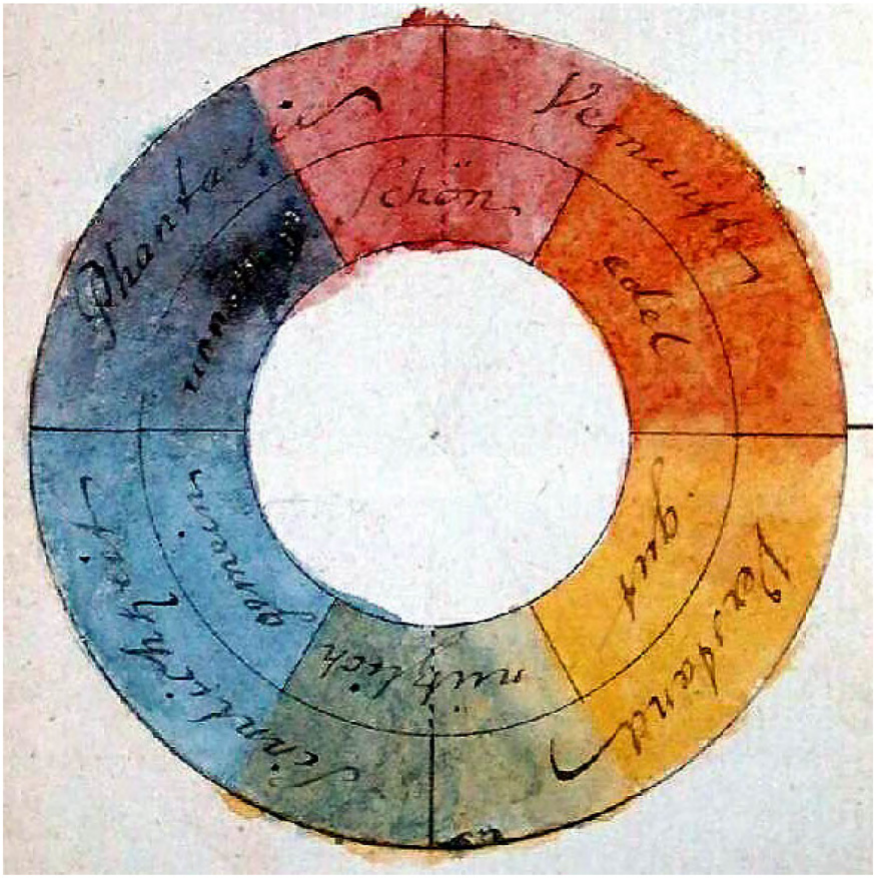
Johann Wolfgang von Goethe, *Farbenkreis zur Symbolisierung des menschlichen Geistes–und Seelenlebens,* 1809 (Original: Freies Deutsches Hochstift–Frankfurter Goethe-Museum). The scheme illustrates the chapter *Allegorischer, symbolischer, mystischer Gebrauch der Farbe* in Goethe’s *Farbenlehre*. One has: (inner circle) red: schön; orange: edel; yellow: gut; green: nützlich; blue: gemein; purple: unnöthig. (outer circle) red–orange: Vernunft; yellow–green: Verstand; green–blue: Sinnlichkeit; purple–red: Phantasie. It is a six-step division that merges red and magenta and splits yellow in orange and yellow (proper). (Of course, colors may have changed over the course of two centuries.)

One should not forget that the color circle as a strong Gestalt has other implications than abstract color science. The version drawn by Goethe (1810) (Figure 20) may serve to illustrate this.^16^ The book by Philip Otto Runge (1810), which superficially looks like a scientific text, is mostly about religious interpretations and implications. Modern texts by artists as Paul Klee (2023), Johannes Itten (1974), and Vassily Kandinsky (1911) are also to the point. Rudolf Arnheim (1974) has a good section on this topic.

Here the affective values and relations to other realms (Kandinsky: *Musikalisch dargestellt ist helles Blau einer Flöte ähnlich*) are predominant. Kandinsky was a well known sound-color synesthete. Such associations are common enough. Remember Locke’s (1690) story:

A studious blind man, who had mightily beat his head about visible objects, and made use of the explication of his books and friends, to understand those names of light and colors which often came in his way, bragged one day, That he now understood what scarlet signified. Upon which, his friend demanding what scarlet was? The blind man answered, It was like the sound of a trumpet. Just such an understanding of the name of any other simple idea will he have, who hopes to get it only from a definition, or other words made use of to explain it.

This is rather different from Goethe, whose associations are in the cognitive domain. In æsthetics proper (Baumgarten, 1750) one deals with the affective logic of (sensory) intuitions, whereas in cognition one deals with the formal, Fregian logic of reasoning. There appears to be a large, gray area in between.

The “well-tempered color circle” is evidently in the æsthetic domain, whereas the most commonly used color circles in “color pickers” (Koenderink et al., 2016) are based on an engineering RGB account (not to be confused with the CIE RGB system (CIE, 1932)) that has only recently been related (Koenderink et al., 2021a) to the core-colorimetry of Maxwell (1855) and Helmholtz (1855). Thus we consider this work “experimental phenomenology” (Albertazzi et al., 2015), rather than vision science.

## Notes

1. For instance, Ostwald uses an odd mix of formal and empirical methods. His method of mensuration by the “Principle of Internal Symmetry” is well described by Bouma (1946).

2. Thus, Ostwald’s concept of “color, white and black content” has survived in computer graphics, although its origin has been forgotten and the nomenclature suggests something else. The common hsv and hsl systems use “chroma” like Ostwald’s *Farbton* and introduce various qualities (“value,” “lightness,” “saturation,” etc.) that are just Ostwald’s *Farb–*, *Weiss–*, and *Schwarz–gehalt* in various repackaged forms. The latter are just algebraic combinations of the numbers {0,min(R,G,B),max(R,G,B),1} . Use of hsv or hsl systems essentially extends the Ostwald system to modern times without anybody noticing. One of the founders of computer graphics, Alvy Ray Smith (who created hsv) reinvented the Ostwald system (Smith, 1996). His abstract is: The two most common color selector models, other than RGB (Red-Green-Blue), are the hue-based HSV (Hue-Saturation-Value) and HSL (Hue-Saturation-Lightness) color models. It is shown that both of these models are flawed. A closely related model, HWB (Hue-Whiteness-Blackness), is introduced that avoids the flaws, is slightly faster to compute, and is very easy to teach to new users: Choose a hue. Lighten it with white. Darken it with black. That whitening is not the complement of blackening is explained. Thus Smith reinvented the wheel, for this is just Ostwald’s system. But Smith was not aware of the (huge) German (also Polish, Russian, Dutch, and Scandinavian) literature.

3. It is an academic exercise, not necessarily of much use. We intend to bring the underlying principles and conventions to attention. In the present implementation you do not have an explicit difference. You are forced to compare two instances in memory. For all practical purposes the standard rygcbm circle of computer graphics remains the most useful.

4. If we say “nonsense” we mean that ontologically distinct concepts or objects are strictly imcomparable. Comparing the proverbial “apples and oranges” is generally understood as nonsensensical. Thus the term is meant in a technical, not pejorative sense.

5. Graßmann actually published a “mathematical proof” that Helmholtz’s observation was wrong, but he departed from the notion that Newton was always right.

6. The symmetry derives from the fact that spectral albedo is limited to 0%–100%. Thus a color A with spectral albedo $s_{A}(\lambda )$ and a color B with spectral albedo $s_{B}(\lambda )=1-s_{A}(\lambda )$ are mutually “supplementary,” since they add to white. For each object color there exists a supplementary one, thus the color solid must be centrally symmetric. It also implies that the object color gamut is a finite volume. This is an essential difference from the space of colors of beams. Due to the constraints on spectral albedo object color space has a much tighter structure than the Maxwell–Helmholtz color mixture space.

7. Remember that the chromaticity diagram is a projective plane, so you can’t directly compare distances. Comparisons should be done in color space, or—for display units—in rgb–space. The conventional chromaticity diagram tends to be very misleading.

8. “Monochromatic object colors are *black*, because they scatter no radiant power at all.”

9. Colorimetric space is a linear space, but not a Hilbert space. In the literature, one often tacitly assumes the latter though (e.g., Cohen & Kappauf (1982). A metric in this sense, essentially an inner product, should not be confused with what are generally referred to as “color metrics.” There have been early attempts at (Riemann) metrics, for instance by Helmholtz (1891) and Schrödinger (1920b, 1920c, 1920d). However, this did not lead to a generally accepted account. Nowadays, one makes do with conventional approximations defined by the CIE.

10. Examples are the HSV, HSL, CIELCH-uv, and CIELCH-ab models. Just Google the Internet for examples.

11. The boundaries are limited by the spectral limits and their complementaries. The transitions are either mutually complementary, or have one “transition” at a spectral boundary. There are four types, namely red to yellow, yellow to cyan, cyan to blue and blue (over purple) to red.

12. Not to be confused with industry claims based on bit-depth. Thus 8-bit RGB yields $(2^{8}{)}^{3}=16,777,216$ distinct colors in the RGB cube volume! The issue of whether these would all look different never comes up—it wouldn’t sell as well, buyers like larger numbers.

13. The “busy backgrounds” are needed to put the colored patches in context. This makes them proper “object colors,” even when presented on a display unit. When displayed on a uniform black background, the colors would be “unrelated.” Unrelated colors are different from object colors in that they do not contain grays, browns, olives and so forth.

14. We encountered one participant that scored on the 0.01 level, although scoring normal on the Ishihara test. Since this participant apparently responded effectively at random—for no apparent reason—we left it out of the analysis as an apparent outlier. This is where the rank correlation comes in handy, in generic cases it is ignored.

15. Our dichromatic observers tend to resolve more like 20 hues, less than the lowest value found for generic (trichromatic) observers.

16. This figure also illustrates how powerful the graphical discretization works. Here you are forced to follow Goethe’s eye, it is very hard to impose Klee’s eye on it. Comparing color circles only makes sense when such arbitrary discretizations are absent. But the eye finds it very hard to compare smooth gradients, it almost “cries out” for a discrete scale.
